# The relationship between callous-unemotional traits and internalizing psychopathology in adolescent psychiatric inpatients: a network analysis

**DOI:** 10.1186/s13034-024-00853-6

**Published:** 2024-12-27

**Authors:** Laura Maria Derks, Eni Sabine Becker, Wolf-Gero Lange, Mike Rinck, Anna Lena Dapprich, Martin Holtmann, Tanja Legenbauer

**Affiliations:** 1 Department for Child and Adolescent Psychiatry, Psychosomatic and Psychotherapy, LWL University Hospital of the Ruhr University Bochum, Heithofer Allee 64, 59071 Hamm, Germany; 2 Behavioural Science Institute, Program of Experimental Psychopathology and Treatment, Radboud University Nijmegen, Nijmegen, The Netherlands; 3 Present Address: Karakter Child and Adolescent Psychiatry University Centre Nijmegen, Nijmegen, The Netherlands

**Keywords:** Callous-unemotional traits, Internalizing behavior, Adolescence, Depression, Anxiety, Network analysis

## Abstract

**Background:**

Numerous studies have investigated the relevance of callous-unemotional traits in relation to externalizing psychopathology among children and adolescents. However, less research has examined the connections between callous-unemotional traits and internalizing psychopathology and findings were inconsistent. Consequently, the present study aimed to elucidate the role of callous-unemotional traits in the context of depression and anxiety while controlling for conduct problems, age, and gender.

**Methods:**

The study utilized self-report questionnaire data from 978 adolescent psychiatric inpatients (*M*_*age*_ = 15.18, *SD* = 1.44) presenting a range of psychopathological conditions. A network analysis was conducted, incorporating callous-unemotional traits, depressive symptoms, anxiety symptoms, conduct problems, and covariates (age, gender). Additionally, comparisons were made between the networks of inpatients diagnosed with conduct disorders and those with internalizing disorders.

**Results:**

The findings indicated that callous-unemotional traits were relevant within the general network, as well as in both the conduct disorder and internalizing networks. In both contexts, callous-unemotional traits were predominately positively associated with depression and conduct problems. Within the conduct disorder network, callous-unemotional traits exhibited primarily negative associations with anxiety, whereas the relationships within the internalizing network were more varied.

**Conclusions:**

Our findings suggest that callous-unemotional traits hold substantial relevance for internalizing symptoms, supporting the notion that these traits should be considered potentially transdiagnostic factors.

**Supplementary Information:**

The online version contains supplementary material available at 10.1186/s13034-024-00853-6.

## Highlights

- The link between CU-traits and internalizing psychopathology is inconsistent.
- A CU-internalizing-externalizing network was constructed for adolescent psychiatric inpatients.
- The networks of CD patients and INT patients differed; however, in both networks, CU-traits played a central role.
- CU-traits have a complex relationship with anxiety, and CU-facets are not a uniform risk or protective factor for anxiety.

**Supplementary Information:**

The online version contains supplementary material available at 10.1186/s13034-024-00853-6.

## Introduction

The relevance of callous-unemotional (CU) traits (i.e., deficits in empathy, lack of guilt and remorse, indifference toward one’s own performance, and lessened or lack of affect [1]) for externalizing psychopathology (e.g., oppositional-defiant disorder, conduct disorder [CD]) in adolescents has been studied extensively over the past decades. Research has established numerous associations with disruptive behavior symptoms. For example, elevated CU-traits predict greater severity and persistence of delinquency and aggression, involvement with the criminal justice system, substance abuse, and animal cruelty in youth [2–5].

In contrast, studies focusing on the relevance of CU-traits for internalizing psychopathology (e.g., depression, anxiety) during childhood and adolescence are less numerous than for externalizing psychopathology. Moreover, they often yield inconsistent findings. With respect to anxiety, for example, some studies have reported that increased fearless behavior and lower levels of trait anxiety are related to CU-traits (e.g., [6, 7]), whereas other studies have reported no correlation between CU-traits and anxiety levels (e.g., [8, 9]), or even positive correlations (e.g., [10]). When controlling for conduct problem (CP) severity, most studies revealed a negative association between CU-traits and anxiety levels (e.g., [7]). Some studies found a moderating effect of CU-traits on associations between externalizing disorders and anxiety levels. Children and adolescents with externalizing disorders and higher CU-traits exhibited lower anxiety levels than those with externalizing disorders and lower CU-traits. They also had less anxiety than children and adolescents with other clinical disorders [11]. Frick and Ray [12] summarize a growing body of research showing that even the high CP and high CU-group cannot be considered homogeneous. They proposed that different developmental pathways lead to differences in anxiety scores. According to the authors, this yields two groups: Children and adolescents with high CU-traits and high levels of anxiety versus children and adolescents with high CU-traits and low levels of anxiety.

A limited number of studies exist with respect to the relationship between CU-traits and depression. For example, in a 10-year follow-up study, mood disorders in childhood predicted CU-traits in adulthood [13]. CU-traits also showed a negative relationship with ratings of suicidality [14]. In a recent study, elevations in conduct problems [CP] and CU-traits over the course of a year during adolescence were associated with elevations in levels of anxiety and depression [15]. In female adolescents, CU-traits, and especially the unemotional subscale, were positively correlated with social phobia, depression, and internalizing symptoms generally in a school sample but not in a clinical sample [16]. Moreover, female adolescents with both high anxiety and high CU-traits presented more significant symptoms of depression [17].

An explanation for the conflicting results, particularly regarding anxiety and CU-traits, may lie in the multidimensionality of CU-traits. Factor analyses of the Inventory of Callous-Unemotional Traits (ICU; [18]), often used to measure CU-traits, have identified three CU subdimensions: uncaringness (i.e., indifference towards other people’s feelings and performance on essential tasks), unemotionality (i.e., deficient emotional affect), and callousness (i.e., lacking empathy and remorse). A meta-analytic review of the validity of the ICU found that the uncaringness and callousness subscales reliably correlate with psychopathic traits generally and with externalizing behaviors, such as aggression, delinquency, and hyperactivity [19]. In contrast, the unemotional subscale showed weak or no correlations to externalizing behaviors, psychopathy, and the other two subscales, suggesting that it may be less relevant to these constructs. These findings indicate that different dimensions of CU-traits may be differentially associated with externalizing and internalizing symptoms. Most studies treat CU-traits as a unidimensional construct by relying on the ICU total score, which obscures whether elevated CU-traits are primarily driven by the unemotional subscale or by the callous or uncaring subscales. This methodological approach may help explain the inconsistent findings in previous research.

Another aspect of the multidimensionality of CU-traits refers to at least two distinct aetiological pathways underlying the presence of CU-traits: a primary and a secondary pathway. Primary CU-traits are thought to be present from birth. They are associated with diminished socioemotional responses to distress (i.e., hypoarousal), fearless temperament, and deficits in emotional processing [20]. Consequently, the relationship between primary CU-traits and internalizing symptoms should be negative, particularly between primary CU-traits and anxiety. In contrast, secondary CU-traits are believed to develop during childhood and adolescence as a possible response to adverse events and environmental factors (e.g., trauma, maltreatment, low parental warmth, and environmental adversity) [21, 22]. Patients with secondary CU-traits are characterized by hyperarousal, in contrast to hypoarousal, and increased sensitivity to socioemotional stimuli. This hyperarousal may then trigger negative affect and lead to higher rates of internalizing symptoms, such as depression and anxiety [20]. It is also possible that in patients who have experienced many adverse life events, CU-traits and internalizing symptoms develop comorbidly as a response to these experiences. Secondary CU-traits could explain results from studies finding positive associations and comorbidities between internalizing symptoms (e.g. depression and anxiety) and CU-traits (e.g., [15, 16]). CU-traits could then be regarded as a transdiagnostic phenomenon relevant across the spectrum of internalizing and externalizing symptoms. The concept of CU-traits as a potential transdiagnostic construct also fits well within the framework of network theory. Network analysis can test the proposed associations between CU-traits and internalizing symptoms. It can also investigate possible transdiagnostic mechanisms of CU-traits.

### Network theory

Within network theory, a disorder is regarded as a cluster of functionally related symptoms. These symptoms are not caused by a latent variable but rather constitute the disorder itself [23, 24]. More specifically, symptoms of a certain disorder (e.g., depression) are regarded as nodes (e.g., anhedonia, depressed mood) within the network and are directly related to each other via edges (e.g., the relationship between anhedonia and depressed mood). In a real-world translation, network theory proposes that when a sufficient number of symptoms are activated for a sufficient amount of time, activation spreads across the network, resulting in the manifestation of the disorder [24]. Typically, it is hypothesized that symptoms within a certain disorder are related to each other; however, some symptoms exhibit greater centrality to the network (i.e., they have more and stronger connections to the other symptoms). These central symptoms can be regarded as the core symptoms of the disorder that have a high impact on the network. Consequently, (de)activating them, for example, by trauma or psychotherapy, should result in the (de)activation of the disorder [24, 25]. Network theory further proposes that there are symptoms that do not clearly belong to a distinct disorder but rather show connections to the symptom networks of different mental disorders. These bridge symptoms (e.g., rumination) create overlap between different mental disorders by displaying edges to one disorder (e.g., depression) and another disorder (e.g., anxiety) and, when activated, are thought to explain the pathway that underlies comorbidity [23]. Within network theory, bridge symptoms can then be regarded as transdiagnostic factors [25], and CU traits could be one of these transdiagnostic factors for internalizing and externalizing psychopathology.

### CU-networks

Network analysis has been applied several times to study the architecture of CU-traits as well as associations with psychopathology. Most studies employed the ICU [18] to measure CU-traits. Two studies assessed the network structure of the ICU among children and adolescents. Bansal and colleagues [26] constructed a CU-network within a group of preschool children (*M*_*age*_ = 4.76) in which more than two-thirds had a diagnosis of an externalizing disorder. The most central items in their network belonged to the callousness dimension. Deng and colleagues [27] compared the CU-networks of juvenile offenders (*M*_*age*_ = 17.14) and community youths (*M*_*age*_ = 10.82). Their results also identified callousness items to be most central in both groups and unemotional items to be peripheral. However, psychopathology was not assessed in their study and there was a significant difference in age between the groups: Whereas juvenile offenders were, on average, in late adolescence, community youth were in late childhood. Since adolescence is a critical period for developmental trajectories of CU-traits [20], this age difference might have confounded the results. Two studies focused on the associations between CU-traits and externalizing psychopathology. Bansal and colleagues [28] again investigated a sample of preschool children (*M*_*age*_ = 4.76) in which more than two-thirds had a diagnosis of an externalizing disorder. A combined network with symptoms of ODD, CD, and CU-traits was constructed. The results revealed that an irritable mood, argumentative behavior, aggression, and callousness were the primary bridges for ODD, CD, and CU-traits. Goulter and Moretti [29] investigated associations between CU-traits and CD via network analysis with children and adolescents (*M*_*age*_ = 13.98) suffering from serious behavioral and social-emotional problems. Again, the results showed callousness items to be most central to the network and an important bridge between CD and CU. In sum, within the field of externalizing psychopathology, callousness seems to be the most important factor contributing to the comorbidity between behavioral problems and CU-traits. All network analyses with developmental samples within the field of CU-traits focused on externalizing psychopathology and did not include internalizing symptoms; only the study by Goulter and Moretti [29] included youth suffering from internalizing and externalizing psychopathology. In adults, three network analyses have assessed associations between psychopathic traits and internalizing symptoms. Zhang and colleagues [30] investigated links between psychopathy, anxiety, and depression in a sample of adult male offenders. They found anxiety and depression to be peripheral in the network. Associations with psychopathy facets arose within the affective, behavioral, and antisocial domains. Oba and colleagues [31] explored associations between primary and secondary psychopathy and externalizing and internalizing symptoms within an adult community sample. They found primary psychopathy (more closely resembling CU-traits) to be negatively associated with trait anxiety and secondary psychopathy to be positively related to trait anxiety. Li et al. [32] examined the interrelations among Machiavellianism, narcissism, psychopathy, and depression in university students. Their results showed that psychopathy exhibited high centrality within the network and had significant relations to suicidal ideation. These studies provide preliminary evidence for the potential relevance of CU-traits to internalizing psychopathology in adults. Due to the inconsistencies in studies regarding the role of CU-traits in internalizing symptoms in childhood and adolescence, it remains unclear whether CU-traits should be addressed in the treatment depression and anxiety, or whether they are more relevant for externalizing disorders in these age groups. This is particularly relevant for adolescence, a period marked by a heightened risk for developing internalizing symptoms [33]—however, most network analyses have been based on child or adult samples.

With regard to the associations between anxiety and depression in adolescence, to our knowledge, three network analyses have been conducted in samples with clinical levels of psychopathology [34–36]. Results showed that depression and anxiety were closely connected. The studies found the following bridges between the two constructs: restlessness or trouble relaxing, sad mood and lack of cheerful emotions, excessive crying, low self-esteem, disturbed appetite, school impairments, physical symptoms of depression, and worrying.

### Present study

The primary aim of the present study was to identify central symptoms and bridge symptoms in a combined network of CU-traits, internalizing psychopathology (i.e., depression, anxiety), and externalizing psychopathology (i.e., CP) in an adolescent clinical sample while controlling for age and gender as covariates. With respect to the heterogeneity of findings in the studies that investigate the relationship between CU-traits and internalizing symptoms, the network analysis is thought to be hypothesis-generating rather than hypothesis-testing. Our second aim was to test, using a network comparison test [37], whether the aforementioned network would differ significantly between adolescents with CDs and adolescents with internalizing disorders (INT). We expect that the networks of CD- and INT-patients differ significantly with respect to global strength invariance and centrality invariance. We hypothesize that the network of CD-patients is denser than the network of INT-patients, indicating stronger associations between CU-traits, internalizing, and externalizing symptoms in CD than in INT. Moreover, we expect CU- and CP-nodes to be more central in the CD-network and depression and anxiety nodes to be more central in the INT-network. With respect to other network comparisons (i.e., network structure, edge invariance), no specific hypotheses can be formulated owing to the lack of studies in this area.

## Materials and methods

### Participants

Data from 1002 adolescent inpatients with varying psychiatric symptomatology of the LWL-University Hospital for Child and Adolescent Psychiatry Hamm in Germany were used for the study. Patients who had a schizophrenia spectrum disorder or other psychotic disorders that would affect study participation were excluded (*n* = 24). The final sample consisted of 978 inpatients between 12 and 18 years of age. The sample was predominantly female, and the majority of participants had comorbidities. The most common main diagnoses were depressive disorders, followed by CDs and substance abuse disorders. The most common comorbid diagnoses were substance abuse disorders (*n* = 191), emotional disorders with onset specific to childhood (*n* = 132), anxiety disorders (*n* = 115), CDs (*n* = 114), and depressive disorders (*n* = 105). Table 1 presents an overview of the demographic and clinical characteristics of the sample.

**Table 1:** Sample characteristics

Characteristic	*M*	*SD*
Age	15.18	1.44
No. of diagnoses	2.23	1.40
Treatment duration in days^a^	53.31	35.61
General psychopathology	18.32	5.84
CU-traits	26.67	9.43
Depression	14.62	6.91
Anxiety	45.24	21.44
Conduct problems	2.69	1.97

	*n*	*%*
Female	658	67.3
Primary diagnosis^b^		
Depressive disorders	493	50.4
Conduct disorders	170	17.4
Substance abuse disorders	120	12.3
Other^c^	195	19.9
Comorbidities	626	64.0

*N* = 978. General psychopathology and CPs were measured with the SDQ-Deu, CU-traits with the ICU, depression with the PHQ-9, and anxiety with the SCAS-D.

^a^Treatment duration refers to the time between hospital admission and discharge in days.

^b^Diagnoses were based on the ICD-10. Only diagnoses with a frequency above 10% in the current sample are reported in the table.

^c^Other primary diagnoses included neurotic, stress-related and somatoform disorders (*n* = 76), behavioural and emotional disorders with onset usually occurring in childhood and adolescence (other than conduct disorders; *n* = 53), behavioural syndromes associated with physiological disturbances and physical factors (*n* = 46), disorders of psychological development (*n* = 9), disorders of adult personality and behaviour (*n* = 8), and mood disorders (other than depression; *n* = 3).

### Measures

#### Demographic variables

To better characterize the study sample, demographic variables (i.e., age, gender, psychiatric diagnoses, and duration of inpatient treatment) were retrieved from the participants’ digital hospital records.

#### CU-traits

CU-traits were measured with the German self-report version of the Inventory of Callous-Unemotional Traits (ICU; [18]). The ICU consists of 24 items (see Table S.1) that are scored on a 4-point Likert scale ranging from 0 (not at all true) to 3 (definitely true). The items describe indifference in relation to one's own performance and feelings of others, lack of guilt, empathy, and remorse, and an absence of emotional expression. The ICU consists of three subscales with good psychometric properties: namely, Callousness, Uncaring, and Unemotional, and initial support has been obtained for its validity [38, 39]. By adding all the items, a total score can be created. In the current study, Cronbach’s α was 0.83 for the total score, 0.72 for the unemotional subscale, 0.78 for the uncaring subscale, and 0.74 for the callousness subscale.

#### Depression

Depression was measured with the German version of the Patient Health Questionnaire-9 (PHQ-9; [40]). It consists of nine items (see Table S.1) that correspond to the nine DSM-IV-TR criteria for Major Depressive Disorder [41]. The self-reported items are rated on a 4-point Likert scale ranging from 0 (not at all) to 3 (nearly every day) and refer to the occurrence of depressive symptoms over the last two weeks. The PHQ-9 has proven to be a valid tool for measuring depression in adolescents [42]. In the current study sample, Cronbach’s α was 0.88 for the total score.

#### Anxiety

Anxiety was measured with the German version of the Spence Children’s Anxiety Scale (SCAS-D; [43]). The SCAS-D is a self-report questionnaire that measures anxiety levels based on six different subscales, i.e., generalized anxiety, panic/agoraphobia, social phobia, separation anxiety, obsessive–compulsive disorder, and physical injury fears. The 38 items (see Table S.1) are rated on a 4-point Likert scale ranging from 0 (never) to 3 (always). The SCAS-D total and subscale scores showed excellent validity and internal consistency in previous research [44]. In our sample, Cronbach’s α was 0.94 for the total score, 0.81 for the general anxiety subscale, 0.88 for the panic/agoraphobia subscale, 0.85 for the social phobia subscale, 0.68 for the separation anxiety subscale, 0.78 for the obsessive–compulsive disorder subscale, and 0.60 for the physical injury fears subscale.

#### Conduct problems

CPs were measured with the CP-subscale of the German version of the Strength and Difficulties Questionnaire (SDQ-Deu; [45]). The self-report version of the SDQ-Deu measures psychopathology in children on four subscales (i.e., CPs, hyperactivity-inattention, emotional symptoms, and peer problems) with five items each, rated on a 3-point Likert scale ranging from 0 (not true) to 2 (certainly true). A total difficulties score can be derived by adding up the subscale scores. In the current study, the CP-subscale was used to measure CP, and the total difficulties score was used to measure participants’ level of general psychopathology. A previous German validation study reported satisfactory validity of the self-reported total score in a clinical setting, whereas the validity of the CP-subscale was low [46]. In our sample, Cronbach’s α was 0.60 for the CP-subscale and 0.75 for the total score.

### Procedure

The data used in the current study were derived from the psychiatry’s routine diagnostic assessment, which includes a self-report questionnaire battery and takes approximately one hour. Patients who were admitted to the hospital between September 2020 and January 2023 and who completed the ICU, the SASC-D, the PHQ-9, and the SDQ-Deu as part of the standard assessment were included in the study. The local medical-ethical committee approved the use of these data for study purposes (Ruhr University Bochum, No. 4359–12).

### Data preparation and statistical analysis

The data were prepared, and descriptive statistics, chi^2^-, and t-tests were performed using IBM SPSS Statistics (version 29.0.0.0). Network analyses and network comparison test were conducted using R (version 4.2.2) and RStudio (RStudio 2023.06.1).

### Network analysis 1

#### Node selection

Before constructing the network, the *goldbricker* function [47] within the *networktools* package was used to identify redundant nodes. Redundant nodes were defined as nodes that differed in less than or equal to 25% of their correlation to other nodes in the network. Following the algorithm’s proposal, eight items were removed from the network: two CU-items (i.e., CU8, CU24) and six anxiety items (i.e., A2, A5, A12, A19, A22, A33).

#### Network construction

The regularized partial correlation network of CU-traits, depression, anxiety, CPs, and covariates was estimated using the *glasso* function within the *qgraph* package [48] combined with extended Bayesian Information Criterion (EBIC) model selection [49]. A tuning parameter (γ) of 0.25 was chosen to achieve a moderately conservative trade-off between including false positive edges and removing true edges and to increase specificity and interpretability [50]. Spearman correlation was applied because the data were skewed and the use of polychoric correlation resulted in a dense network. Centrality indices (betweenness, closeness, strength, expected influence; [51]) were calculated with the *qgraph* functions *centralityPlot* and *centralityTable*.

#### Delta network construction

The impact of covariates on the connections among the clinical variables was examined by creating a delta network. The delta network was constructed by subtracting the network constructed with covariates from the same network constructed without covariates. This delta network then displays the remaining edges between the clinical variables that are due to the impact of the covariates.

#### Identifying bridges

Bridge items between CU-traits, anxiety, depression, and CPs in the network were identified with the *bridge* function of the *network tools* package [47]. Bridge strength and bridge expected influence can be calculated to measure which item of one community (i.e., CU-traits [CU], depression, anxiety, conduct problems [CP]) is most strongly connected to all items in the other three communities and vice versa. Bridge strength is defined as the sum of the absolute values of all edges between a particular node in one community and all the nodes of the other communities. Bridge expected influence is defined as the sum of the partial correlations of a node with all nodes that are not in the same community and, in contrast to bridge strength, accounts for negative edges. Bridge expected influence can be used to assess a node’s influence on immediate neighbors in other communities, and a higher bridge expected influence value indicates a greater bridge influence on neighboring communities [51].

#### Stability and accuracy analyses

The stability of the network was tested with the *case-dropping* function of the *bootnet* package [52]. Edge weight stability, the stability of all centrality indices and the centrality stability coefficients (SCs) for the network as well as for bridge parameters were calculated. An SC above 0.25 and preferably above 0.50 was considered a precondition for interpreting centrality, in line with Epskamp and colleagues [52].

### Network analysis 2

#### Group creation and node selection

Two groups were created to compare network differences between CDs and INTs. The CD-group (*n* = 280) included all patients diagnosed with CD. The INT-group (*n* = 512) included all patients with a main diagnosis of anxiety or depressive disorder and no comorbid CD. Because the current network analysis only used a subsample (*n* = 792) of the one used in network analysis 1 (*N* = 978), we repeated the *goldbricker* analysis [47]. This time, following the algorithm’s proposal, ten items were removed from the network: two depression items (i.e., D4, D5) and eight anxiety items (i.e., A2, A5, A18, A19, A22, A32, A33, A36).

#### Network construction

Two separate regularized partial correlation networks for the CD- and INT-groups with the items of CU-traits, anxiety, depression, CPs and the two covariates were estimated. The networks were constructed via the same method as in network analysis 1.

#### Network comparison test

A network comparison test between the CD- and INT-networks was performed with the *NetworkComparisonTest* package [37]. The network comparison test can be applied to test for differences in network structure (i.e., is the way the nodes are connected different across the two samples), differences in global strength (i.e., is the sum of the strength of all edges combined different across samples), differences in centrality indices (i.e., do different items occupy critical positions in the network across samples), and differences in edge strength (i.e., is the strength of a specific edge different across samples).

### Transparency statement

The current project was preregistered on aspredicted.org (https://aspredicted.org/1VZ_LV6). After receiving peer-review, the data analysis plan was adapted accordingly, and a new preregistration was created prior to data analysis on osf.io (https://doi.org/10.17605/OSF.IO/DCJS4).

## Results

### Network analysis 1

#### Stability analyses

The stabilities of edge weights, expected influence, and strength were excellent (*SCs* = 0.75, see Figure S.1 for the edge stability graph and Figure S.2 for the centrality stability graph). The stabilities of closeness and betweenness were good (*SCs* = 0.52). The stabilities for bridge strength and bridge expected influence were excellent (*SCs* = 0.75).

#### Network of CU, depression, and anxiety

**Fig. 1:**
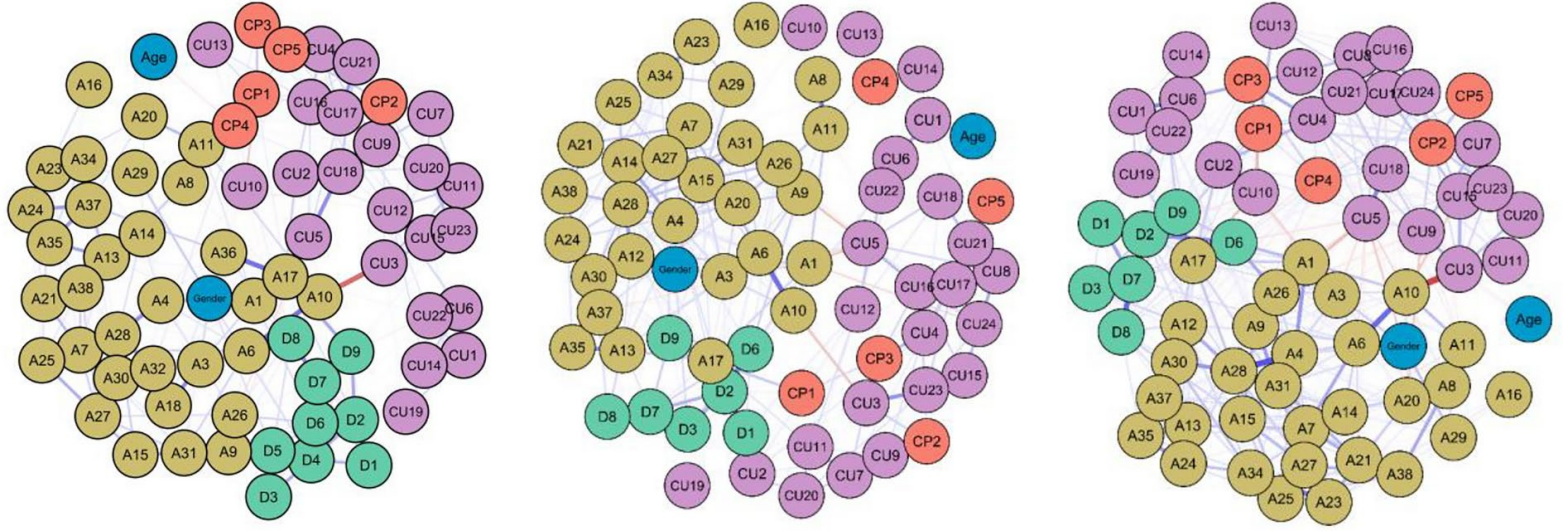
Networks of the full sample, the INT-subsample, and the CD-subsample. Left panel: network across the whole sample (*n* = 978), middle panel: network of the INT-group (*n* = 512), right panel: network of the CD-group (*n* = 280). CU-traits are in purple, anxiety symptoms are in yellow, depressive symptoms are in green, CPs are in red, and covariates are in blue. Network plots were created with the *plot* function of the *networktools* package [47] and the layout type *spring*, which uses the Fruchterman-Reingold force-directed algorithm. Nodes are arranged based on their connections. Nodes with more and/or stronger connections are positioned closer together. Thicker lines between nodes represent stronger relationships. Blue lines represent positive associations, red lines represent negative associations.

The network (Fig. 1) included 70 nodes (see Table S.1 for a description of each questionnaire node): 22 CU-nodes, nine depression nodes, 32 anxiety nodes, five CP-nodes, and two covariates (i.e., age, gender).

Since the centrality estimates of betweenness, closeness and strength were substantially interrelated (*r* ≥ 0.63), we focused our interpretation on strength centrality to increase readability and interpretability. Expected influence correlated strongly with strength (*r* = 0.67) but only weakly with betweenness (*r* = 0.18) and closeness (*r* = 0.34) and was therefore reported separately. Whereas strength centrality is calculated by adding up the absolute values of all edges connected to a certain node, expected influence takes the relative values of all connected edges to a node and, therefore, accounts for negative edges within a network.

**Fig. 2:**
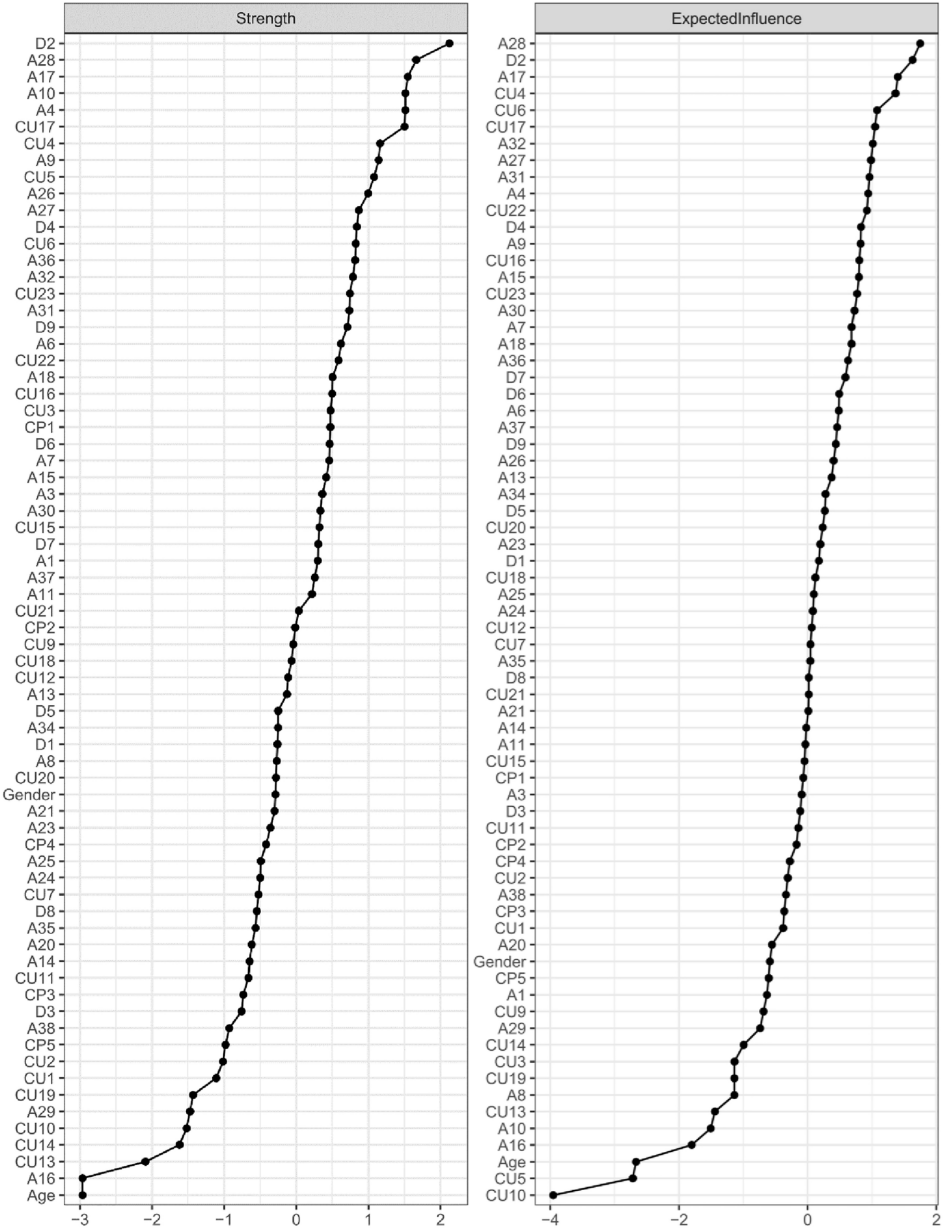
Strength and expected influence centrality plots. Strength and expected influence centrality plots. z-scores were used as the scale on the x-axis. Higher values indicate that a node is more central to the network.

As shown in Fig. 2, the five nodes with the greatest node strength centrality were D2 [depressed mood] (2.12), A28 [sudden fear] (1.66), A17 [obsessive thoughts] (1.55), A10 [worry about school performance] (1.51), and A4 [feeling afraid] (1.51), whereas the two least central nodes were A16 [fear of dogs] (− 2.96) and Age (− 2.96). The five nodes with the greatest expected influence were A28 [sudden fear] (1.75), D2 [depressed mood] (1.63), A17 [obsessive thoughts] (1.40), CU4 [uncaring about hurting others] (1.36), and CU6 [not showing emotions] (1.08), whereas the two nodes with the least influence were CU5 [feeling guilty*] (− 2.72) and CU10 [not letting feelings take control] (− 3.96).

### Impact of covariates on the relationships among CU-traits, internalizing psychopathology, and externalizing psychopathology

The impact of age and gender on the connections among clinical variables was examined by creating a delta network. The delta network had very few edges, with a mean edge weight of 0.01 and a maximum edge weight of 0.02. The sum of the edges of the 68 symptoms was reduced from 31.6 to 30.7 by controlling for age and gender, which means that including the covariates accounted for only 2.7% of the connectivity between clinical variables in the network. This was emphasized by the remarkably high correlation between the networks with and without covariates (*Spearman’s ρ* = 0.98). Consequently, for the bridge symptom analysis, the two covariate nodes, age and gender, were removed from the network to increase readability and interpretability.

#### Bridges between CU, depression, and anxiety

**Fig. 3:**
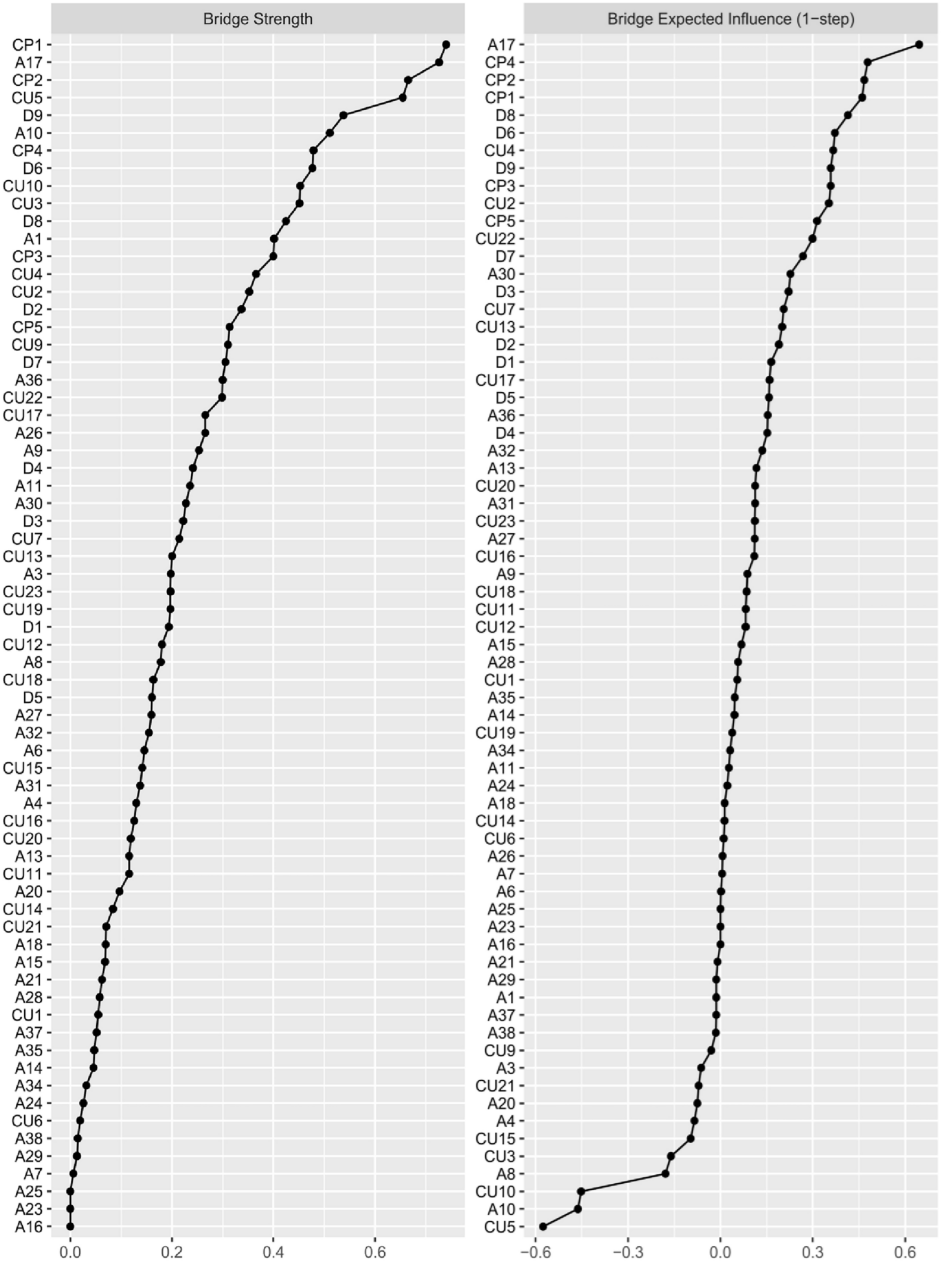
Bridge strength and bridge expected influence centrality plots. Bridge strength and bridge expected influence centrality plots. Higher values indicate that a node is more central to the network.

Figure 3 shows the bridge strength and bridge expected influence of the network.

#### Bridge strength

In the *CP-*cluster, CP1 [losing temper] had the greatest bridge strength (0.74) and was most strongly linked to CU10 [not letting feelings take control] (part *r* = − 0.12), A17 [obsessive thoughts] (part *r* = 0.06), and D7 [concentration problems] (part* r* = 0.04) in the other communities. In the *anxiety* cluster, A17 [obsessive thoughts] had the greatest bridge strength (0.73) and had the strongest associations with D9 [suicidal ideation] (part *r* = 0.19), CU2 [not knowing right and wrong] (part *r* = 0.12), and CP1 [losing temper] (part *r* = 0.06) in the other communities. In the *CU-*cluster, CU5 [feeling guilty*] (0.65) had the greatest bridge strength and correlated most strongly with A1 [worrying] (part *r* = − 0.08), D2 [depressed mood] (part* r* = − 0.05), and CP2 [obedience*] (part *r* = 0.04). In the *depression* cluster, D9 [suicidal ideation] had the greatest bridge strength (0.54) and was most strongly connected to A17 [obsessive thoughts] (part *r* = 0.19) and CU22 [hide feelings] (part *r* = 0.06) in the other two communities and had no associations with conduct problems (all *r*s = 0.00).

#### Bridge expected influence

In the *anxiety* cluster, A17 [obsessive thoughts] also had the greatest bridge expected influence (0.64). In the *CP-*cluster, CP4 [lying/cheating] had the greatest bridge expected influence (0.48) and was most strongly linked to A32 [sudden heart pounding] (part *r* = 0.07), CU18 [lack of remorse] (part *r* = 0.06), and D8 [psychomotor functioning] (part *r* = 0.06) in the other two communities. In the *depression* cluster, D8 [psychomotor functioning] had the greatest bridge expected influence (0.41) and was most strongly linked to A36 [intrusive thoughts or pictures] (part *r* = 0.08), CP4 [lying/cheating] (part *r* = 0.06), and CU2 [not knowing right and wrong] (part *r* = 0.04). In the *CU-*cluster, CU4 [uncaring about hurting others] had the greatest bridge expected influence (0.37) and correlated most strongly with CP3 [fighting/forcing others] (part *r* = 0.18), A13 [compulsive behavior] (part* r* = 0.02), and D8 [psychomotor functioning] (part *r* = 0.01).

### Network analysis 2

#### Descriptive statistics

Table 2 summarizes the sample differences between the CD- and INT-groups in terms of sample characteristics and questionnaires. The samples were significantly different in terms of age, gender, comorbidities, and psychopathological burden. The INT-sample was older, included more females, and had higher scores for general psychopathology, depression, and anxiety. The CD-sample had more comorbidities, more CU-traits, and more CPs. More specific diagnostic information about the subsamples can be found in Tables S.2a and S.2b in the supplement.

**Table 2:** Characteristics and differences for the groups of network analysis 2

Characteristic	CD (*n* = 280)	INT (*n* = 512)	*t*	*Cohen’s d*
	*M (SD)*	*M (SD)*		
Age	15.06 (1.49)	15.28 (1.41)	2.095^*^	0.156
Treatment duration	51.10 (42.00)	50.89 (25.31)	0.075	0.006
No. of diagnoses	3.17 (1.74)	1.71 (0.84)	13.202^***^	1.180
General psychopathology	17.36 (6.26)	19.58 (5.11)	5.086^***^	0.401
CU-traits	29.76 (9.91)	25.90 (8.82)	5.444^***^	0.419
Depression	11.04 (6.43)	17.26 (5.95)	13.656^***^	1.015
Anxiety	34.94 (19.94)	52.46 (19.63)	11.944^***^	0.888
Conduct problems	3.73 (2.01)	2.27 (1.77)	10.172^***^	0.785

	*n (%)*	*n (%)*	*χ*^*2*^	*Cohen’s d*
Female	104 (37.1)	416 (81.3)	161.728^***^	1.011

*Note. *Group differences were tested with t- or chi^2^-tests.

^***^p ≤ 0.001 ^**^p ≤ 0.01 ^*^p ≤ 0.05

#### Stability analyses

In the *CD-group,* the stabilities of strength, edge weights, and expected influence were acceptable (*SCs* = 0.36), whereas the stabilities of closeness and betweenness were poor^1^ (*SCs* = 0 and 0.13, respectively). In the *INT-group*, the stabilities of strength, edge weights, and expected influence were good (*SC*_*strength*_ = 0.67, *SC*_*edge*_ = 0.67, *SC*_*expected influence*_ = 0.59), the stability of betweenness was acceptable (*SC* = 0.36), and the stability of closeness was poor (*SC* = 0).

#### Comparison of the networks of CD-patients and INT-patients

**Fig. 4:**
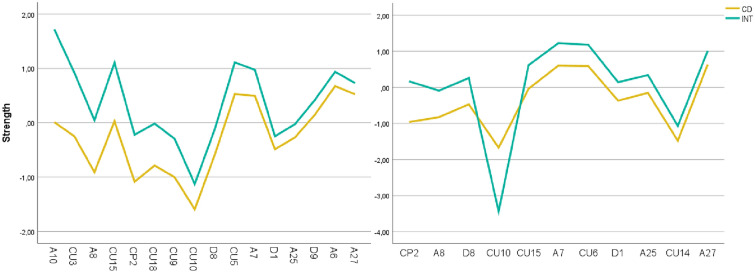
Significantly different strength centrality values of the INT- and CD-groups. Significantly different nodes for the INT- (*n* = 512) and CD-groups (*n* = 280) regarding centrality. Strength centrality in the left panel and expected influence in the right panel. Variables were sorted according to the extent of the difference between samples from left to right.

The results of the network comparison test revealed that the CD- and INT-networks (Fig. 1) were significantly different with respect to *network structure invariance* (*M* = 0.21, *p* = 0.041) as well as with regard to *global strength invariance* (*S*_*difference*_ = 8.18, *p* = 0.026), with a greater global strength in the INT-group (*S* = 27.11) than in the CD-group (*S* = 18.93). Concerning *centrality invariance*, the network comparison test revealed a significant difference (*p* < 0.05) in strength centrality for 16 of the 68 variables (see Fig. 4, left panel): six CU-nodes, six anxiety nodes, three depression nodes, and one CP- node. Expected influence differed significantly for 11 variables (see Fig. 4, right panel)—four CU-nodes, four anxiety nodes, two depression nodes, and one CP-node. All variables had a greater strength and all but one variable had a greater expected influence in the INT-network. Only CU10 [Not let feelings take control] had a greater expected influence in the CD-network. With respect to *edge invariance*, there was a significant difference (*p* < 0.05) between the groups for 75 (3.3%) of the 2278 edge pairs tested. Of those 75 nodes, 31 node pairs contained CU-nodes (41.3%). We focused our interpretation on edge changes between CU-nodes and nodes from other communities. Fifteen significant edge differences arose between a CU-node and a node from another community (see Table S.3). Notably, most differences arose between CU and anxiety (10 nodes), followed by depression (3 nodes), CPs (1 node), and covariates (1 node).

## Discussion

The present study applied network analysis to conceptualize the role of callous-unemotional (CU) traits in internalizing psychopathology in adolescent psychiatric inpatients. In step one, a network of CU-traits, internalizing symptoms (i.e., depression, anxiety), and externalizing symptoms (i.e., conduct problems (CP)) was analyzed within 978 inpatients with a range of psychopathological conditions (network analysis 1). In step two, the network was compared between individuals with conduct disorder (CD) and individuals with internalizing (INT) psychopathology (network analysis 2).

### Core symptoms of the CU-INT comorbidity network

This study aimed to identify the most central symptoms within the combined network of CU-traits and internalizing psychopathology. Depressive symptoms (i.e., *depressed mood*), anxiety symptoms (i.e., *sudden fear, feeling afraid*, *obsessive thoughts, worry about school performance*), and CU-symptoms (i.e., *uncaring about hurting others, not showing emotions*) emerged as core symptoms. The centrality of *depressed mood* might be explained by the fact that half of the sample had a diagnosis of depression as the main diagnosis, and depressive symptoms also co-occur frequently with other diagnoses [53].

Surprisingly, *depressed mood* remained the only depression item among the most central items, whereas anxiety items played an especially prominent role. This might be explained by the high degree of comorbidity between depression and anxiety disorders [54] and the transdiagnostic nature of questionnaire items. Whereas *sudden scare* and *feeling afraid* are typical symptoms of anxiety disorders, *worrying about school performance,* as well as *obsessive thoughts* are rather transdiagnostic symptoms. For example, worrying about school might also result from concentration problems and lower performance levels typically associated with depression [55] or substance abuse disorders [56] or from disruptive behavior problems typically occurring in CD [57]. Although the item regarding *obsessive thoughts* is meant to measure obsessive thoughts occurring in obsessive–compulsive disorder, the item is framed in rather general language. Thus, depressive patients might experience suicidal thoughts as obsessive, whereas, for example, youth with substance abuse might experience thoughts about taking drugs as obsessive. Worrying or rumination about a certain topic might, therefore, be a transdiagnostic symptom rather than a pure anxiety symptom.

Rather surprisingly, two CU-items were among the most central nodes. The first CU-item, *not showing emotions*, points to a connection between the unemotionality facet of CU-traits and internalizing symptoms. Within (primary) CU-traits, unemotionality is regarded as a trait present from birth and is already observable in 6-month-old babies [58], accompanied by deviations at the neural level (e.g., reduced amygdala activity toward emotional stimuli; [59]). In this concept, unemotionality refers not only to expressing emotions but also to not experiencing emotions as strongly (e.g., lessened threat reactions [6] and reduced affective empathy [3]). The item, however, measures not showing emotions more generally. Youths with depression [60, 61], social phobia (e.g., [62]) and CU-traits [1] all seem to have diminished emotional expressions; however, the reasons underlying this facet of unemotionality possibly differ strongly between the diagnostic categories. Whereas adolescents with social phobia might show less emotional expression to avoid interaction and due to an internal state of fear [63], diminished expression in depressed individuals may serve the purpose of inhibiting ongoing emotional reactivity in adverse and potentially dangerous environments [64]. Thus, not expressing emotions can be regarded as a transdiagnostic symptom that occurs in diverse psychopathologies, and this transdiagnostic mechanism could potentially help explain the results of studies finding positive associations between measures of CU-traits and anxiety and depression [15, 16].

The relevance of the second central CU-item, *uncaring about hurting others,* seems counterintuitive at first glance. Although correlations with other CU-symptoms and with CPs seemed likely, associations with depressive and anxiety symptoms were rather unexpected. An explanation might be the concept of a secondary variant of CU-traits [20]. In patients who experienced a high number of adverse life events, CU-traits and internalizing symptoms may have developed comorbidly in response to adverse experiences. In patients with primary CU-traits, however, no associations or negative associations with internalizing symptoms might arise. Independent of their origin (primary or secondary pathway), the CU-traits expressed by patients are theoretically thought to be the same (for conflicting evidence, see [65]). The ICU, which was employed in the present study, cannot differentiate between the variants. Yet, the high number of internalizing diagnoses in the total sample (i.e., more than half of the participants had a primary diagnosis of depression) makes the presence of secondary CU-traits more likely. These secondary CU-traits might drive correlations between CU-traits and anxiety or depressive symptoms, resulting in a relatively high centrality of CU-symptoms.

### Transdiagnostic mechanisms between anxiety, depression, and CU-traits

In the network analysis, *losing temper* and *lying/cheating* emerged as relevant bridge symptoms, or transdiagnostically relevant symptoms, in the CP-cluster. Within the anxiety cluster, *obsessive thoughts* were the most important bridge item. Within the depression cluster, *psychomotor functioning* and *suicidal ideation* were the most important bridges. With respect to CU-traits, *uncaring about hurting others* was also a bridge item, together with feeling guilty*. CU-traits did not have substantially lower bridge values than the other clusters did. The bridge strength value was even greater for some CU-items than for depression items. This result seems to provide evidence for a potentially transdiagnostic mechanism of CU-traits. With respect to CPs, CU-traits seem to be a risk factor: There were positive bridges between not feeling guilty* and obedience* and between *uncaring about hurting others* and *fighting/forcing others*, which fits well with the literature [3]. For anxiety and depressive symptoms, the picture was more complex. Feeling guilty* seemed to be a protective factor as it has negative bridge associations with *worrying* and *depressed mood*. This finding is consistent with the literature showing that guilt is positively associated with internalizing disorders and negatively associated with externalizing disorders [66]. In contrast, *uncaring about hurting others* may be a risk factor for the activation of *psychomotor functioning* and *checking behavior* through positive bridges. One reason for this might be the hyperarousal found in adolescents with high CU-traits and high comorbid internalizing psychopathology (i.e., secondary CU-variant) [20]. *Checking behavior* might be a strategy to cope with hyperarousal and associated stronger responses to socioemotional stimuli. *Psychomotor functioning* captures slowing down or being restless. Restlessness might also be a symptom of the hyperarousal associated with secondary CU-traits.

### Comparisons of the CD- and INT-networks

The results of the network comparison between the CD- and INT-subsamples were contrary to our expectations. We expected the CD-network to be denser than the INT-network, as indicated by strength invariance. We also expected CD and CP nodes to be especially central in the CU-network, and depression and anxiety to be especially prominent in the INT-network, expressed by centrality invariance. Instead, our results all pointed in one direction: CU-traits were relevant in both networks. Moreover, the INT-network had a greater density (i.e., greater connectivity among symptoms) than the CD-network. Based on the literature, one would expect that the higher density scores might be explained by stronger edges between anxiety and depressive symptoms owing to their symptomatic overlap and comorbidity [53] in internalizing samples. However, most of the variables that were significantly more central in the INT-network were CU-nodes. These CU-items included symptoms from all three CU-facets: unemotionality, callousness, and uncaringness. This surprising finding fits well with the model of secondary CU-traits and is in line with previous findings of increased anxiety and depression in this patient group [15]. Interestingly, CD seemed to have an impact on these relations: Within the CD-network, CU-symptoms exhibited mostly positive associations with CPs and depression symptoms but negative associations with anxiety symptoms. This matches the pattern associated with primary CU-traits (e.g., [4]). In contrast, in the INT-network, only a few (mostly negative) pathways were observed between CU-traits and anxiety symptoms, which shows that CU-traits and anxiety might be unrelated in INT-samples. This was also reflected in the edge invariance test that pointed to significant differences among CU-internalizing pathways between the networks. The results are consistent with the contradictory findings in the literature that have established positive associations between CU- and internalizing symptoms (e.g., [15]), no associations (e.g., [9]), or negative associations (e.g., [4]). The data further show that depression and anxiety should not be combined when looking at associations with CU-traits.

### Translation to psychopathology and psychotherapy

Our results contribute to the current understanding of the architecture and transdiagnostic role of CU-traits. CU-items were central and influential not only in the CD-network but also in the INT-network and the overall transdiagnostic network—even when controlling for CPs, age, and gender. Interestingly, items from the unemotional facet of CU-traits were not the only ones associated with internalizing symptoms. Items from the callousness and uncaringness dimensions of CU-traits also served as bridges between the disorders. This finding extends the results from previous network analyses that identified these dimensions as bridges to externalizing symptoms [28, 29], highlighting their relevance for internalizing symptoms as well.

The network comparison pointed to somewhat differential associations of CU-traits and internalizing symptoms between the CD- and INT-patient groups. These divergent results might be related to differences in CU-variants: primary and secondary CU-traits, respectively. Whereas the CD-network might have contained a higher proportion of participants characterized by primary CU-traits, the INT-network might have contained a higher proportion of participants with secondary CU-traits. As the ICU used in this study to measure CU-traits does not account for the aetiology of CU-traits, further studies are needed to shed light on this hypothesis. In sum, our results point to the relevance of CU-traits across diagnostic categories in line with the previous work of Herpers and colleagues [67], rather than having relevance only for externalizing behavior problems, as proposed by Frick and Moffitt [68]. Clinically, our results have implications for psychotherapeutic approaches within CU-traits. CU-traits are traditionally only targeted in the treatment of externalizing patient groups; however, our results imply that it might be helpful to address CU-traits in the treatment of internalizing patients. There is accumulating evidence (for a review, see [20]) that adolescents who are high on both CU-traits and internalizing psychopathology are at higher risk for adverse outcomes. Therefore, studies should investigate the effects of including CU-traits in treatment programs for internalizing patients.

Comorbidities traditionally present a challenge for clinicians because they have a negative impact on treatment success, and it is often unclear which symptoms should be prioritized during treatment. Central and bridge symptoms might be a reasonable choice to address first in treatments because targeting them may disrupt pathways that maintain comorbidity and spread across several symptoms [24, 69]. Our results suggest that when combining results regarding node and bridge centrality, clinicians should focus especially on the anxiety item *obsessive thoughts*. This item was among the items with the highest (bridge) centrality and was the only node in the network nominated in all categories. Furthermore, no difference in the centrality of this node between the subsamples was found, indicating an equally high relevance of this item in both subgroups. As mentioned above, it remains unclear whether the high centrality of *obsessive thoughts* is due to the wording of the item, which might capture very different types of thoughts experienced as obsessive (e.g., suicidal or negative thoughts in depression, thoughts about substances in addiction, or thoughts about a specific anxiety-related stimulus in phobic disorders). If the item captures worrying or rumination, as speculated, then the current results match results from another network analysis in an adolescent clinical sample: in Ruan et al.’s [35] network analysis, worrying was established as an essential bridge between anxiety and depression. Further studies are needed to investigate whether *obsessive thoughts* are also central to other symptom networks in adolescent psychiatric samples.

### Limitations

The present study has several limitations. The sample was not equally balanced across the categories of anxiety, depression, CPs, and CU-traits. Whereas approximately half of the sample had a primary diagnosis of depression, less than one-fifth of the participants had a primary diagnosis of CD, and only a small number of participants suffered primarily from an anxiety disorder. Additionally, our sample was highly comorbid and had a high level of psychopathology in general, as we recruited psychiatric inpatients. The study has been conducted in Germany, which might have implications on the sample's composition in terms of the most prevalent diagnostic categories. Symptoms were assessed via self-reports, which might be more reliable for internalizing (depression, anxiety) than for externalizing (CP, CU) symptoms [70] and might be biased by the participants’ current emotional state. Future studies should include a more balanced sample regarding internalizing and externalizing disorders. Symptoms should be assessed with multi-informant reports.

The data collected in the study are cross-sectional. Therefore, we cannot make any statements regarding the causality and predictive value of the results. The identified bridge and core symptoms may be influenced by other symptoms, actively influence other symptoms, or both within the causal chain. Future studies should assess symptoms longitudinally to obtain a clearer understanding of developmental trajectories among symptoms.

The groups compared with the network comparison test had unequal group sizes. Therefore, the results for the INT-group have more statistical power than those for the CD-group. Future studies should recruit more patients for both groups. Finally, we cannot generalize our findings across cultures or age groups, as this was not a multicenter international study that spans the lifespan.

## Conclusions and future directions

The present study sheds light on the nature of the comorbidity between CU-traits and internalizing symptoms and contributes to a currently understudied field of research. Our findings suggest a complex interplay between CU-traits and internalizing psychopathology. Future studies should further investigate the transdiagnostic potential of CU-traits and clarify whether transdiagnostic mechanisms of CU-traits only emerge in cross-sectional research or whether these associations can be observed in longitudinal studies. For example, future studies should shed light on the predictive validity of CU-traits for internalizing and externalizing symptoms and for the development of individual symptom networks over time. Future clinical research should investigate the effectiveness and practicability of targeting suggested bridges and core symptoms identified via network analysis in intervention studies.

## Supplementary Information

Supplementary Material 1.

## Data Availability

No datasets were generated or analysed during the current study.

## Abbreviations

CU-traits: Callous-unemotional traits
CD: Conduct disorder
ICU: Inventory of callous-unemotional traits
CP: Conduct problems
INT: Internalizing subgroup of the current sample
PHQ-9: Patient Health Questionnaire-9
SCAS-D: Spence Children’s Anxiety Scale-Deutsch
SDQ-Deu: Strength and Difficulties Questionnaire-Deutsch
SC: Stability coefficient

## Footnotes

1 A poor SC indicates that a measure cannot be estimated reliably. Small changes to the data could lead to substantial differences in the results. Therefore, the measure should not be interpreted.

## Code availability

The software used is referenced in the text.
