# Supplementary material for: The relationship between callous-unemotional traits and internalizing psychopathology in adolescent psychiatric inpatients: a network analysis
Source: Child Adolesc Psychiatry Ment Health. 2024 Dec 27;18:163. doi: 10.1186/s13034-024-00853-6 (PMC11681694; doi:10.1186/s13034-024-00853-6)
Supplement: Supplementary file 1 — Supplementary Material 1. [file 13034_2024_853_MOESM1_ESM.docx]

**Supplementary Material**

**Figure S.1**


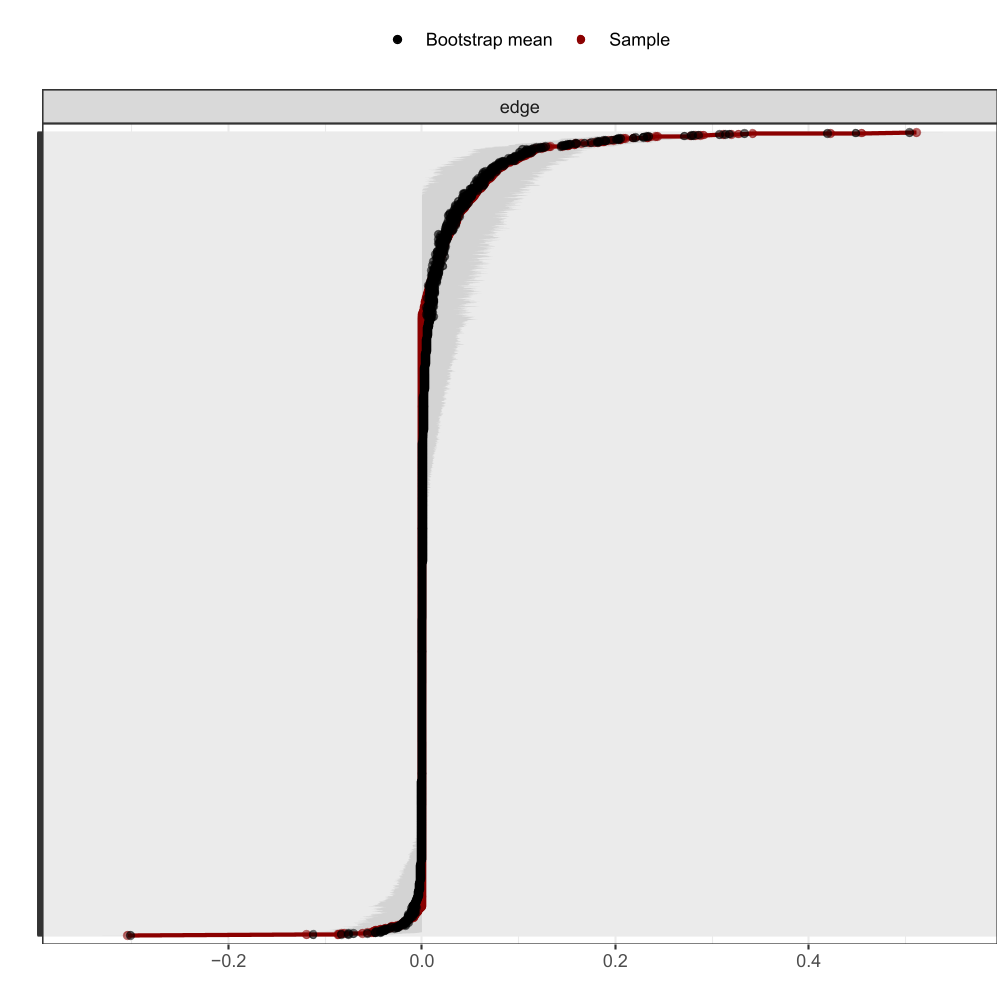
*Edge Weight Stability in Network Analysis 1*

*Note.* Bootstrapped confidence intervals of estimated edge-weights for the estimated network 1. The *red line* indicates the sample values and the *gray area* indicates the bootstrapped CIs. Each *horizontal line* represents one edge of the network, ordered from the edge with the highest edge-weight to the edge with the lowest edge-weight. The y-axis labels have been removed to avoid cluttering**.**

**Figure S.2**


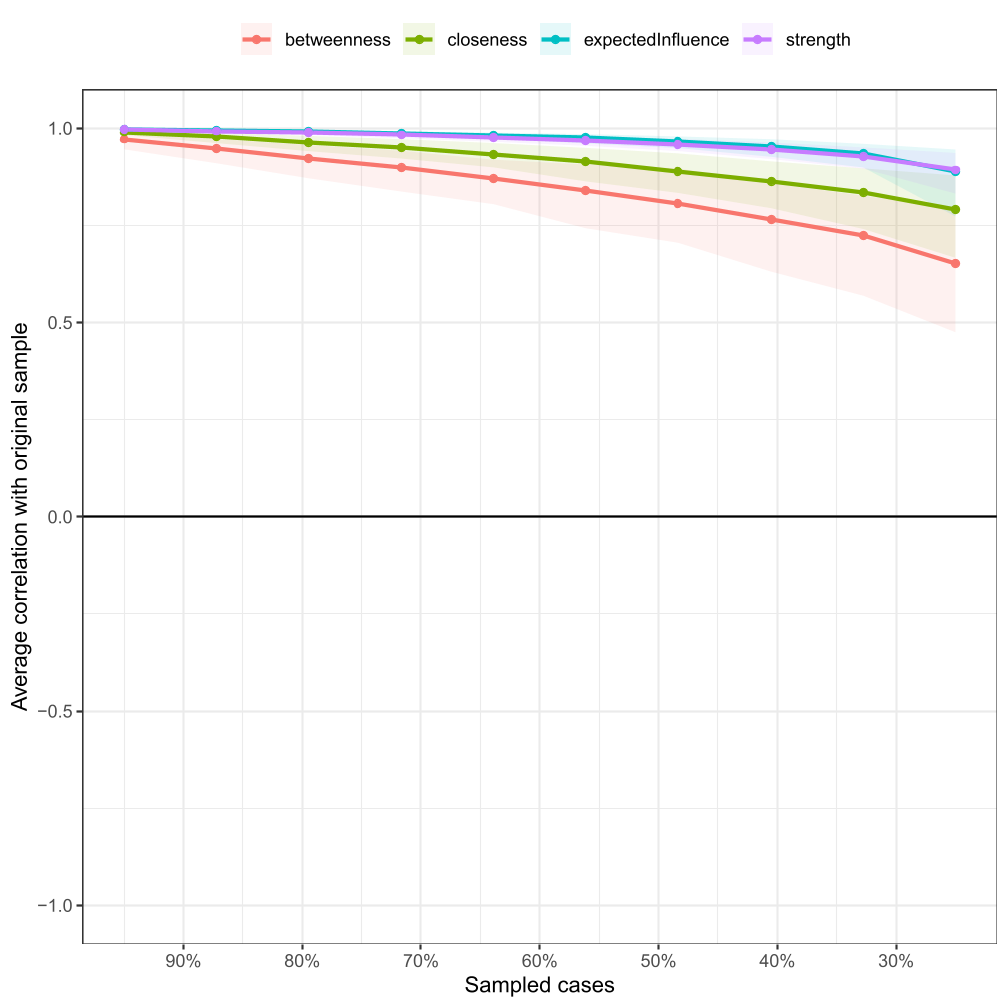
*Centrality Stability in Network Analysis 1*

*Note.* Average correlations between centrality indices of networks sampled with persons dropped and the original sample. *Lines* indicate the means and *areas* indicate the range from the 2.5th quantile to the 97.5th quantile.

**Table S.1**

*Nodes in the Network*

| Node | Questionnaire Item | Abbreviation |
| --- | --- | --- |
| ICU Unemotional Subscale | | |
| CU1* | I express my feelings openly. | Express feelings |
| CU6 | I do not show my emotions to others. | Not showing emotions |
| CU14* | It is easy for others to tell how I am feeling. | Others can see emotions |
| CU19* | I am very expressive and emotional. | Emotionally expressive |
| CU22 | I hide my feelings from others. | Hide feelings |
| ICU Callousness Subscale | | |
| CU2 | What I think is “right” and “wrong” is different from what other people think. | Not knowing right and wrong |
| CU4 | I do not care who I hurt to get what I want. | Not caring to hurt others |
| CU7 | I do not care about being on time. | Not on time |
| CU8* | I am concerned about the feelings of others. | Concern for other’s feelings |
| CU9 | I do not care if I get into trouble. | Uncaring about trouble |
| CU10 | I do not let my feelings control me. | Not letting feelings take control |
| CU11 | I do not care about doing things well. | Uncaring about performance |
| CU12 | I seem very cold and uncaring to others. | Cold and uncaring |
| CU18 | I do not feel remorseful when I do something wrong. | Lack of remorse |
| CU20 | I do not like to put the time into doing things well. | Does not like investing time into doing things well |
| CU21 | The feelings of others are unimportant to me. | Unimportance of other’s feelings |
| ICU Uncaring Subscale | | |
| CU3* | I care about how well I do at school or work. | Care for school performance |
| CU5* | I feel bad or guilty when I do something wrong. | Feeling guilty |
| CU13* | I easily admit to being wrong. | Admit to being wrong |
| CU15* | I always try my best | Try best |
| CU16* | I apologize (“say I am sorry”) to persons I hurt. | Apologize |
| CU17* | I try not to hurt others’ feelings. | Not wanting to hurt others |
| CU23* | I work hard on everything I do. | Work hard |
| CU24* | I do things to make others feel good. | Make others feel good |
| PHQ-9 | | |
| D1 | Little interest or pleasure in doing things. | Anhedonia |
| D2 | Feeling down, depressed, or hopeless. | Depressed mood |
| D3 | Trouble falling or staying asleep, or sleeping too much. | Sleep problems |
| D4 | Feeling tired or having little energy. | Fatigue |
| D5 | Poor appetite or overeating. | Appetite problems |
| D6 | Feeling bad about yourself — or that you are a failure or have let yourself or your family down. | Worthlessness |
| D7 | Trouble concentrating on things, such as reading the newspaper or watching television. | Concentration problems |
| D8 | Moving or speaking so slowly that other people could have noticed or the opposite - being so fidgety or restless that you have been moving around a lot more than usual. | Psychomotor functioning |
| D9 | Thoughts that you would be better off dead or of hurting yourself in some way. | Suicidal ideation |
| SASC-D General Anxiety | | |
| A1 | I worry about things. | Worrying |
| A3 | When I have a problem, I get a funny feeling in my stomach. | Funny stomach feeling |
| A4 | I feel afraid. | Feeling afraid |
| A18 | When I have a problem, my heart beats really fast. | Pounding heart |
| A20 | I worry that something bad will happen to me. | Worry about oneself |
| A22 | When I have a problem, I feel shaky. | Feeling shaky |
| SASC-D Social Phobia | | |
| A6 | I feel scared when I have to take a test. | Fear of tests |
| A7 | I feel afraid if I have to use public toilets or bathrooms. | Fear of public bathrooms |
| A9 | I feel afraid that I will make a fool of myself in front of people. | Fear of embarrassment |
| A10 | I worry that I will do badly at my school work. | Worry about school performance |
| A26 | I worry what other people think of me. | Fear of negative evaluation |
| A31 | I feel afraid if I have to talk in front of my class. | Fear of talks |
| SASC-D Panic/Agoraphobia | | |
| A12 | I suddenly feel as if I can’t breathe when there is no reason for this. | Sudden trouble breathing |
| A19 | I suddenly start to tremble or shake when there is no reason for this. | Sudden trembling |
| A25 | I feel scared if I have to travel in the car, or on a bus or a train. | Fear of public transport |
| A27 | I am afraid of being in crowded places. | Fear of crowds |
| A28 | All of a sudden I feel really scared for no reason at all. | Sudden fear |
| A30 | I suddenly become dizzy or faint when there is no reason for this. | Sudden dizziness |
| A32 | My heart suddenly starts to beat too quickly for no reason. | Sudden heart pounding |
| A33 | I worry that I will suddenly get a scared feeling when there is nothing to be afraid of. | Worry about sudden fear |
| A34 | I am afraid of being in small closed places, like tunnels or small rooms. | Claustrophobia |
| SASC-D Obsessive-Compulsive Disorder | | |
| A13 | I have to keep checking that I have done things right. | Checking behavior |
| A17 | I can’t seem to get bad or silly thoughts out of my head. | Obsessive thoughts |
| A24 | I have to think of special thoughts to stop bad things from happening. | Compulsive thoughts |
| A35 | I have to do some things over and over again. | Compulsive behavior |
| A36 | I get bothered by bad or silly thoughts or pictures in my mind. | Intrusive thoughts or pictures |
| A37 | I have to do some things in just the right way to stop bad things happening. | Just right behavior |
| SASC-D Physical Injury Fears | | |
| A2 | I am scared of the dark. | Fear of darkness |
| A16 | I am scared of dogs. | Fear of dogs |
| A21 | I am scared of going to the doctors or dentists. | Fear of doctors |
| A23 | I am scared of being in high places or lifts (elevators). | Fear of heights |
| A29 | I am scared of insects or spiders. | Fear of insects |
| SASC-D Separation Anxiety | | |
| A5 | I would feel afraid of being on my own at home. | Fear of being home alone |
| A8 | I worry about being away from my parents. | Worry about separation from parents |
| A11 | I worry that something awful will happen to someone in my family. | Worry about family |
| A14 | I feel scared if I have to sleep on my own. | Fear of sleeping alone |
| A15 | I have trouble going to school in the mornings because I feel nervous or afraid. | Afraid of school |
| A38 | I would feel scared if I had to stay away from home overnight. | Fear of sleep overs |
| SDQ-Deu Conduct Problems | | |
| CP1 | I get very angry and often lose my temper. | Losing temper |
| CP2* | I usually do as I am told. | Obedience |
| CP3 | I fight a lot. I can make other people do what I want. | Fighting/Forcing others |
| CP4 | I am often accused of lying or cheating. | Lying/Cheating |
| CP5 | I take things that are not mine from home, school or elsewhere. | Stealing |

*Indicates reverse-scored item. All items were inverted before calculating the networks so that a higher score on each items indicates a higher symptom strength.

**Table S.2a**

*Diagnostic Information of the INT-subsample of Network Analysis 2*

|  | INT (n = 512) | |
| --- | --- | --- |
|  | n | % |
| Main Diagnosis |  |  |
| Depressive Disorders | 477 | 93.2 |
| Anxiety Disorders | 35 | 6.8 |
| Secondary Diagnosis^a^ |  |  |
| Anxiety Disorders | 84 | 16.4 |
| Emotional Disorders | 82 | 16.0 |
| Other^b^ | 166 | 32.4 |
| Comorbid Depressive and Anxiety Disorders | 100 | 19.5 |

*Note.* Diagnostic classifications were based on the ICD-10.

^a^ Only diagnoses with a frequency above 10% in the current subsample are reported in the table.

^b^ Other secondary diagnoses included behavioural and emotional disorders with onset usually occurring in childhood and adolescence (other than emotional disorders; *n* = 37), neurotic, stress-related and somatoform disorders (other than anxiety disorders; *n* = 28), disorders of adult personality and behaviour (*n* = 26), behavioural syndromes associated with physiological disturbances and physical factors (*n* = 22), substance abuse disorders (*n* = 20), depressive disorders (*n* = 20), and disorders of psychological development (*n* = 13).

**Table S.2b**

*Diagnostic Information of the CD-subsample of Network Analysis 2*

|  | CD (n = 280) | |
| --- | --- | --- |
|  | n | % |
| Main Diagnosis^a^ |  |  |
| Conduct Disorders | 170 | 60.7 |
| Substance Abuse Disorders | 91 | 32.5 |
| Other^b^ | 19 | 6.8 |
| Secondary Diagnosis^a^ |  |  |
| Substance Abuse Disorders | 138 | 49.3 |
| Conduct Disorders | 114 | 40.7 |
| Hyperkinetic Disorders | 48 | 17.1 |
| Other^c^ | 89 | 31.8 |

*Note.* Diagnostic classifications were based on the ICD-10.

^a^ Only diagnoses with a frequency above 10% in the current subsample are reported in the table.

^b^ Other main diagnoses included mood disorders (*n =* 17) and pervasive developmental disorders (*n* = 2).

^c^ Other secondary diagnoses included behavioural and emotional disorders with onset usually occurring in childhood and adolescence (other than hyperkinetic and conduct disorders; *n* = 29), neurotic, stress-related and somatoform disorders (*n* = 28), disorders of psychological development (*n* = 14), disorders of adult personality and behaviour (*n* = 10), and mood disorders (*n* = 8).

**Table S.3**

*Results of the Edge Invariance Test between the INT- (n = 512) and CD-Groups (n = 280)*

| CU-edge | Paired edge | CD edge (part r) | INT edge (part r) | Edge difference |
| --- | --- | --- | --- | --- |
| CU3 | A10 | -.110 | -.324 | \|.214\|^***^ |
| CU12 | CP2 | .000 | .054 | \|.054\|^*^ |
|  | D1 | .037 | .000 | \|.037\|^*^ |
| CU11 | A17 | .052 | .000 | \|.052\|^*^ |
|  | D2 | .012 | .000 | \|.012\|** |
|  | Gender | .000 | -.004 | \|.004\|^*^ |
| CU5 | A37 | -.045 | .000 | \|.045\|* |
|  | A8 | -.007 | .000 | \|.007\|^**^ |
| CU2 | A37 | .000 | .040 | \|.040\|^*^ |
| CU22 | A7 | .000 | .027 | \|.027\|^*^ |
| CU4 | D9 | .000 | .013 | \|.013\|^***^ |
| CU8 | A27 | .000 | .009 | \|.009\|*** |
|  | A21 | .000 | .002 | \|.002\|^***^ |
| CU16 | A10 | -.008 | .000 | \|.008\|^**^ |
| CU7 | A4 | .000 | -.000 | \|.000\|* |

^***^ *p* < .001 ^**^ *p* < .01 ^*^ *p* < .05
